# MRI-Visible Perivascular Spaces Associated With Cognitive Impairment in Military Veterans With Traumatic Brain Injury Mediated by CSF P-Tau

**DOI:** 10.3389/fpsyt.2022.921203

**Published:** 2022-07-06

**Authors:** Ming-Liang Wang, Dian-Xu Yang, Zheng Sun, Wen-Bin Li, Qiao-Qiao Zou, Peng-Yang Li, Xue Wu, Yue-Hua Li

**Affiliations:** ^1^Department of Radiology, Shanghai Jiao Tong University Affiliated Sixth People’s Hospital, Shanghai, China; ^2^Department of Neurosurgery, Shanghai Jiao Tong University Affiliated Sixth People’s Hospital, Shanghai, China; ^3^Division of Cardiology, Pauley Heart Center, Virginia Commonwealth University, Richmond, VA, United States; ^4^Institute for Global Health Sciences, University of California, San Francisco, San Francisco, CA, United States

**Keywords:** traumatic brain injury, MRI, CSF, tau, Aβ, MRI-visible perivascular spaces

## Abstract

**Objective:**

To investigate the association of MRI-visible perivascular spaces (PVS) with cognitive impairment in military veterans with traumatic brain injury (TBI), and whether cerebrospinal fluid (CSF) p-tau and Aβ mediate this effect.

**Materials and Methods:**

We included 55 Vietnam War veterans with a history of TBI and 52 non-TBI Vietnam War veterans from the Department of Defense Alzheimer’s Disease Neuroimaging Initiative (ADNI) database. All the subjects had brain MRI, CSF p-tau, Aβ, and neuropsychological examinations. MRI-visible PVS number and grade were rated on MRI in the centrum semiovale (CSO-PVS) and basal ganglia (BG-PVS). Multiple linear regression was performed to assess the association between MRI-visible PVS and cognitive impairment and the interaction effect of TBI. Additionally, mediation effect of CSF biomarkers on the relationship between MRI-visible PVS and cognitive impairment was explored in TBI group.

**Results:**

Compared with military control, TBI group had higher CSO-PVS number (*p* = 0.001), CSF p-tau (*p* = 0.022) and poorer performance in verbal memory (*p* = 0.022). High CSO-PVS number was associated with poor verbal memory in TBI group (β = -0.039, 95% CI −0.062, −0.016), but not in military control group (β = 0.019, 95% CI −0.004, 0.043) (*p*-interaction = 0.003). Further mediation analysis revealed that CSF p-tau had a significant indirect effect (β = −0.009, 95% CI: −0.022 −0.001, *p* = 0.001) and mediated 18.75% effect for the relationship between CSO-PVS and verbal memory in TBI group.

**Conclusion:**

MRI-visible CSO-PVS was more common in Vietnam War veterans with a history of TBI and was associated with poor verbal memory, mediated partially by CSF p-tau.

## Introduction

Traumatic brain injury (TBI) is a major cause of morbidity and mortality worldwide, especially in military veterans (1–3). Previous studies have revealed that military veterans with TBI would have a poor performance in cognitive and psychological functioning (4, 5). Epidemiologic Studies even demonstrated an increased incidence of dementia including Alzheimer’s disease (AD) in military veterans with TBI (6, 7). Amyloid beta (Aβ) and/or tau accumulation may play an important role. Recent studies have detected tau and Aβ deposition in the brain of military veterans with TBI by PET examinations (8, 9). However, the upstream mechanisms underlying cognitive impairment remain poorly understood.

MRI-visible perivascular spaces (PVS) are fluid-filled spaces surrounding small penetrating blood vessels (10, 11). Traditionally, MRI-visible PVS was regarded as an imaging marker of small vessel disease and correlated with aging (12). However, more and more studies suggested MRI-visible PVS may be an important imaging marker of dysfunction of brain glymphatic system, through which brain waste like tau and Aβ could be cleared away (13–15). PVS has been proved to be associated with tau pathology (16) and cognitive impairment in Parkinson’s disease (17). In an animal study of TBI, Iliff et al. (18) found chronic impairment of glymphatic system after TBI which may further promote tau aggregation. At present, only a few studies found an increased number of MRI-visible PVS in TBI (19–21). However, the previous studies of TBI did not discriminate CSO-PVS from BG-PVS. It remains unclear about the distribution characteristics of MRI-visible PVS in military veterans with TBI and its association with cognitive impairment and tau and Aβ pathology.

In this study, we hypothesize that (1) military veterans with TBI will have higher MRI-visible PVS number and cerebrospinal fluid (CSF) p-tau and Aβ42/40; (2) MRI-visible PVS would be associated with cognitive impairment, and CSF biomarkers will mediate this effect in military veterans with TBI. Our study would provide an innovative imaging biomarker and a new potential therapeutic target for cognitive impairment in military veterans with TBI.

## Materials and Methods

### Study Subjects

The study data in this study were collected from the DOD-ADNI as part of Alzheimer’s Disease Neuroimaging Initiative (ADNI) database,^1^ which was launched in 2003, led by Principal Investigator Michael W. Weiner, M (22). The primary goal of ADNI project was to test whether serial MRI, PET, other biological markers, and clinical and neuropsychological assessments can be combined to measure the mild cognitive impairment progression and early AD. DOD-ADNI aimed to investigate whether TBI and post-traumatic stress disorder (PTSD) increase the risk for AD in Veteran subjects. Written informed consent was obtained from all the study subjects, and the study protocol was proved by local participating ADNI institutions.

**FIGURE 1:**
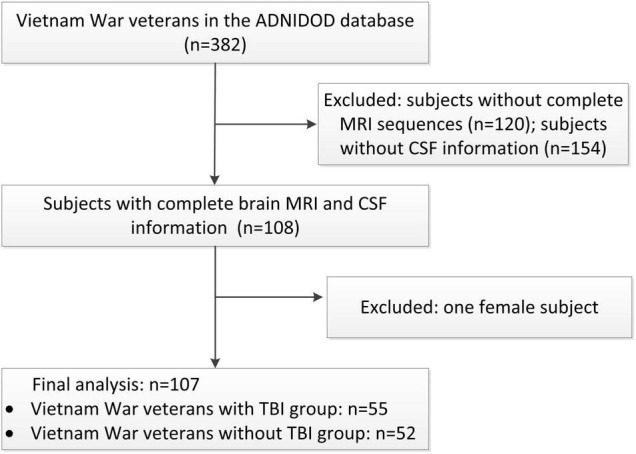
Participants recruitment flow chart.

The inclusion and exclusion procedures were shown in Figure 1. Our study included male Vietnam War veterans with or without a history of non-penetrating TBI, who had complete brain MRI, CSF p-tau, Aβ, and neuropsychological tests results information. Patients with dementia, history of psychosis or bipolar affective disorder, history of schizophrenia (DSM-IV criteria), history of alcohol or substance abuse/dependence within the past 5 years, MRI related exclusions (metal in the body, pacemakers), contraindications for lumbar puncture were excluded in this project. Demographics including age, education, and APOE ε4 status were collected. Medical history of hypertension, diabetes, and hyperlipidemia was also collected from the medical history dataset.

### Traumatic Brain Injury Classification

The documented history of TBI was identified from the Department of Defense or Veterans Administration records, and TBI severity was classified. Mild TBI was defined as loss of consciousness lasting for less than 30 min, and/or a period of post-traumatic amnesia lasting for less than 24 h; moderate-severe TBI was defined as loss of consciousness lasting for at least 30 min, and/or a period of post-traumatic amnesia lasting for at least 24 h (23).

### CSF Biomarker Measurement

CSF samples in this project were analyzed using the ADNI methods to measure the levels of Aβ42, Aβ40, and phosphorylated tau at threonine 181 (p-tau) on a fully automated cobas e601 analyzer (Roche Diagnostics). We especially focused on CSF Aβ42/40 ratio and CSF p-tau, as they were generally thought to reflect Aβ pathology (24) and tau pathology (25).

### Psychiatric and Cognitive Assessment

Lifetime PTSD was recorded using the clinician-administered PTSD scale (CAPS) within DSM-IV (CAPS score > 40). Geriatric depression scale (GDS) test was used to measure depression in older adults. Mini-Mental Status Examination (MMSE) test was used to reflect general cognitive performance. Cognitive tests recommended to assess TBI-related cognitive impairment (26) were selected including Trail Making Test Part A (processing speed), Trail Making Test Part B (executive functioning), Rey Auditory Verbal Learning Test (RAVLT) total score of trials 1–5 (learning), and RAVLT total delayed recall and recognition total (verbal memory). Raw scores of all these cognitive tests were standardized to z-scores for each participant. The average z-scores of RAVLT total delayed recall score and recognition total score were calculated to reflect verbal memory. Higher z-scores indicated poorer performance on the processing speed and executive functioning measures, and lower z-scores indicated poorer performance on verbal learning and verbal memory.

### MRI Data Acquisition and Analysis

All MR examinations were performed according to the ADNI MRI scanning method (GE or Siemens), which included axial T1-weighted, T2-weighted, fluid-attenuated inversion recovery (FLAIR), and T2-star gradient-echo weighted sequences (Supplementary Table 1). Two trained neuroradiologists (M-LW and W-BL with 10 and 24 years of experience, respectively) who were blinded to the clinical and CSF data, assessed PVS, white matter hyperintensities (WMH), and cerebral microbleeds (CMBs) according to the Standards for Reporting Vascular Changes on Neuroimaging (27).

**FIGURE 2:**
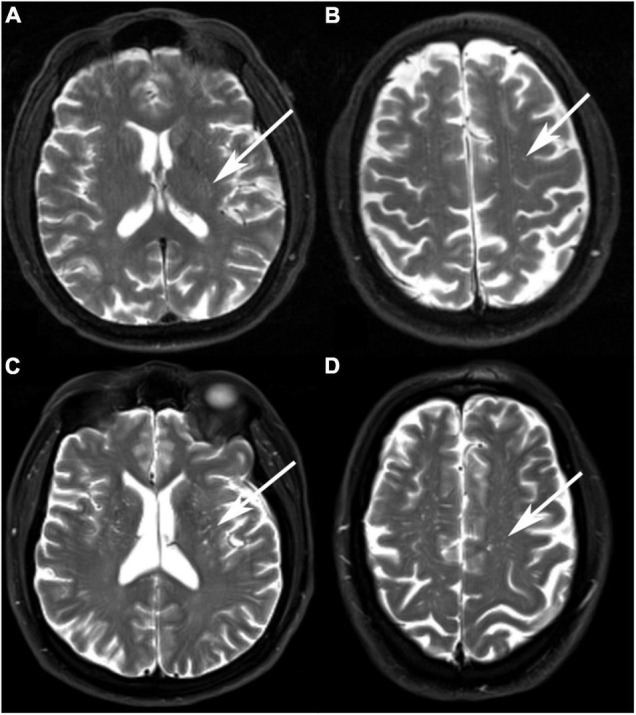
Examples of MRI-visible perivascular spaces. **(A,B)** A 68-year-old Vietnam war veteran without a history of traumatic brain injury (TBI) had a low degree of BG-PVS and CSO-PVS. **(C,D)** A 67-year-old Vietnam War veteran with a history of TBI had a high degree of BG-PVS and CSO-PVS.

Perivascular spaces was assessed by comprehensively reading the T1-weighted, T2-weighted, and fluid-attenuated inversion recovery (FLAIR) sequence and counted specially on T2-weighted sequence. PVS were characterized by locating along the penetrating arteries and shown as low signals on T1-weighted and FLAIR images and high signal on T2-weighted images (Figure 2). PVS was rated in the basal ganglia (BG) and the centrum semiovale (CSO). CSO was defined as white matter superior to the lateral ventricles in each of cerebral hemispheres. The largest number of PVS was documented on one slice of one side of the brain. A validated visual rating scale was also used to assess MRI-visible PVS (0 = no PVS, 1 = 1–10 PVS, 2 = 11–20 PVS, 3 = 21–40 PVS, and 4 = 41 or more PVS) (12, 28). The PVS number and grade were both assessed for analyses.

White matter hyperintensities was evaluated in the periventricular region (PWMH) and deep white matter region (DWMH) according to Fazekas rating scale (29). Severe WMH was defined as having a score > 1 of the Fazekas scale. The total WMH volume was also extracted from the database, and a ln-transform was applied over the data to generate normal distribution data. CMBs, characterized as low signal on the T2 star sequence (30), were visually counted and rated as 0 = microbleeds absent and 1 = microbleeds present.

### Statistical Analyses

Categorical variables were expressed as frequency (percentage) and continuous variables were expressed as mean (standard deviation). The demographics were compared using the chi-square test for qualitative variables and the *t*-test for quantitative variables, as appropriate between TBI and military control group. Analyses of covariance (ANCOVA) was done adjusting for (1) age and APOE ε4 status to examine group differences in CSF biomarkers; (2) age, education, and APOE ε4 status to examine group differences in cognitive performance; (3) age, education, APOE ε4 status, MR scanners (General Electric or Simens), CMBs and lnWMH to examine group differences in MRI-visible PVS number. MRI-visible PVS grade was also analyzed using chi-square test.

Multiple linear regression analyses were applied to estimate the association between MRI-visible PVS and cognitive impairment. The cognitive tests scores were taken as outcome variables with MRI-visible PVS as the predictor. We constructed five models to adjust potential confounding factors: Model 1 was unadjusted; model 2 was adjusted for age, education and APOE ε4 status; model 3 was adjusted for CMBs and lnWMH plus the variables in model 2; model 4 was adjusted for CSF biomarkers plus the variables in model 3. All the analyses were done in TBI group subjects and military control subjects respectively, and the interaction effect of TBI status was explored. Furthermore, as increased PVS may indicate the glymphatic system impairment, which would slow the clearance of brain waste like tau and Aβ and finally cause the occurrence of cognitive impairment, mediation analyses were done to investigate whether CSF biomarkers mediated the association between PVS and cognitive impairment using the SPSS PROCESS module (31, 32). As there were four cognitive scales analyzed in this study, Bonferroni corrected *p*-value was set for 0.05/4 = 0.0125 for the analyses of cognitive scale. *P* < 0.05 was considered as significant for other statistical analyses. All statistical tests were performed by IBM SPSS Statistics for Windows, version 20.0.

## Results

### Demographics and Neuropsychological Function of the Study Population

A total of 55 Vietnam War veterans with a history of TBI (age 68.8 ± 3.8 years, all male) and 52 non-TBI Vietnam War veterans (age 68.3 ± 4.4 years, all male) were included in this study. Compared with military control subjects, TBI group had poorer performance in RAVLT verbal memory composite score (*p* = 0.022) after adjusting for age, education and APOE ε4 carrier status (Table 1). There were no statistical differences in demographics and other neuropsychological tests between military control group and TBI group (*p* > 0.05). Supplementary Table 2 shows the injury characteristics of TBI subjects.

**TABLE 1:** Demographics and neuropsychological tests in Vietnam War veterans with and without a history of traumatic brain injury (TBI).

Characteristics	Military control *n* = 52	TBI *n* = 55	*P-value*
Age (years)	68.3 (4.4)	68.8 (3.8)	0.482
Education (years)	15.2 (2.2)	15.2 (2.4)	0.983
Hypertension, *n* (%)	33 (63.5)	35 (63.6)	0.985
Diabetes mellitus, *n* (%)	18 (34.6)	11 (20.0)	0.089
Hyperlipidemia, *n* (%)	26 (50.0)	30 (54.5)	0.638
PTSD, *n* (%)	21 (40.4)	24 (43.6)	0.733
APOE ε4 carrier, *n* (%)	11 (21.6)	17 (33.3)	0.183
MMSE score	28.5 (1.3)	28.2 (1.7)	0.218
GDS score	2.7 (3.4)	2.3 (2.4)	0.574
RAVLT (learning)	0.0137 (0.932)	−0.0129 (1.068)	0.836
RAVLT (memory)	0.141 (0.753)	−0.133 (0.917)	0.022*
Trails A total time	−0.131 (0.671)	0.124 (1.227)	0.109
Trails B total time	0.0186 (1.002)	−0.0176 (1.007)	0.979

Values are reported as mean (SD) for the continuous variables and as frequency (percentage) for the categorical variables. Data of RAVLT (learning), RAVLT (memory), Trails A total time and Trails B total time were compared with ANCOVA adjusting for age, education, and APOE ε4 status. Other data were compared using the chi-square test for qualitative variables and the t-test for quantitative variables. *p < 0.05 (uncorrected). TBI, traumatic brain injury; PTSD, post-traumatic stress disorder; MMSE, mini mental state examination; GDS, geriatric depression scale; RAVLT, Rey auditory verbal learning test.

### Comparisons of MRI Findings and CSF Biomarkers Between Military Control and Traumatic Brain Injury Group

There were 15 Siemens data (four in military control group and 11 in TBI group) and 92 GE data (48 in military control group and 44 in TBI group) used in our study. The inter-rater reliability was excellent for the CSO-PVS number [intraclass correlation coefficient (ICC) = 0.89, 95% CI: 0.84–0.92], BG-PVS number (ICC = 0.86, 95% CI: 0.81–0.90), CMBs (κ = 0.93, 95% CI: 0.88–0.97), PWMH (κ = 0.86, 95% CI: 0.84–0.90), and DWMH (κ = 0.88,95% CI: 0.83–0.92). The intrarater reliability was determined from a random sample of 50 participants with 1 month interval between the first and second image assessments evaluated by one neuroradiologist. The intra-rater reliability was also excellent for the CSO-PVS number (ICC = 0.92, 95% CI: 0.86–0.95), BG-PVS number (ICC = 0.88, 95% CI: 0.80–0.93), CMBs (κ = 0.96, 95%CI: 0.91–1.00), PWMH (κ = 0.92, 95% CI: 0.87–0.97), and DWMH (κ = 0.91,95% CI: 0.86–0.96). The PVS rating scores of the senior radiologist was used for the final analysis.

Table 2 shows the comparison of MRI findings and CSF biomarkers between military control group and TBI group. Compared with military control group, TBI group had more CSO-PVS both in number (*p* = 0.001) after adjusting age, education, APOE ε4 status, CMBs and WMH and grade (*p* = 0.001). The TBI group also had higher levels of CSF p-tau after adjusting for age and APOE ε4 status (*p* = 0.022). There was no statistical difference in BG-PVS, WMH, and CSF Aβ42/40 (*p* > 0.05).

**TABLE 2:** MRI findings and CSF markers in Vietnam War veterans with and without a history of traumatic brain injury (TBI).

	Military control *n* = 52	TBI *n* = 55	*P-value*
CSO-PVS grade			0.001*
1	21 (40.4)	14 (25.5)	
2	20 (38.5)	12 (21.8)	
3	9 (17.3)	19 (52.7)	
4	2 (3.8)	0 (0)	
CSO-PVS number	14.1 (8.4)	20.3 (9.7)	<0.001*
BG-PVS grade			0.246
1	35 (67.3)	31 (56.4)	
2	17 (32.7)	22 (40.0)	
3	0 (0)	2 (3.6)	
BG-PVS number	7.7 (3.8)	8.0 (4.4)	0.772
Mild PWMH, *n* (%)	32 (61.5)	42 (76.4)	0.538
Severe PWMH, *n* (%)	20 (38.5)	13 (23.6)	
Mild DWMH, *n* (%)	38 (73.1)	43 (78.2)	0.174
Severe DWMH, *n* (%)	14 (26.9)	12 (21.8)	
WMH volume (cm^3^)	5.8 (4.6)	5.0 (7.4)	0.287
CMBs, *n* (%)	16 (30.8)	11 (20.0)	0.200
CSF p-tau	17.3 (7.3)	21.0 (8.1)	0.022*
CSF Aβ 42/40	0.0698 (0.017)	0.0704 (0.017)	0.983

Values are reported as mean (SD) for the continuous variables and as frequency (percentage) for the categorical variables. Data of CSO-PVS number and BG-PVS number were compared with ANCOVA adjusting for age, education, APOE ε4 status, MR scanners (General Electric or Simens), CMBs, and lnWMH. Data of CSF p-tau and CSF Aβ 42/40 were compared with ANCOVA adjusting for age and APOE ε4 status. Other data were compared using the chi-square test for qualitative variables and the t-test for quantitative variables. *p < 0.05. TBI, traumatic brain injury; CSO, centrum semiovale; BG, basal ganglia; PVS, perivascular spaces; PWMH, periventricular white matter hyperintensities; DWMH, deep white matter hyperintensities; CMBs, cerebral microbleeds; CSF, cerebral spinal fluid.

### Multiple Regression Analysis Between MRI-Visible PVS and CSF Biomarkers and Cognitive Impairment

In multiple regression analysis, high CSO-PVS number was associated with poor RAVLT memory even after adjusting for age, education, apo ε4, CMBs, lnWMH and CSF biomarkers in TBI group (β = −0.039, 95% CI −0.062, −0.016), but not in military control group (β = 0.019, 95% CI −0.004, 0.043) (*p*-interaction = 0.003). There was tendency of association between high CSO-PVS number and poor RAVLT verbal learning in TBI group (β = −0.037, 95% CI −0.066, −0.007), but not in military control group (β = 0.015, 95% CI −0.020, 0.050) (Table 3). No significant associations were found between CSO-PVS with TMA and TMB test results (*p* > 0.05).

**TABLE 3:** Multiple linear regression of CSO-PVS and cognitive test results: overall and stratified to traumatic brain injury (TBI) history.

	Model	Military control *n* = 52	*P-value*	TBI *n* = 55	*P-value*	*p-*interaction
		β (95% CI)		β (95% CI)		
RAVLT verbal learning	1	0.018 (−0.013, 0.049)	0.240	−0.041 (−0.069, −0.013)	0.005*	0.006*
	2	0.017 (−0.014, 0.049)	0.270	−0.036 (−0.065, −0.007)	0.017	0.012*
	3	0.020 (−0.013, 0.054)	0.233	−0.044 (−0.072, −0.015)	0.004*	0.016
	4	0.015 (−0.020, 0.050)	0.392	−0.037 (−0.066, −0.007)	0.017	0.047
RAVLT verbal memory	1	0.006 (−0.019, 0.032)	0.606	−0.047 (−0.070, −0.025)	0.001*	0.002*
	2	0.018 (−0.006, 0.042)	0.145	−0.043 (−0.065, −0.020)	0.005*	<0.001*
	3	0.025 (0.000, 0.049)	0.051	−0.048 (−0.071, −0.025)	0.003*	0.001*
	4	0.019 (−0.004, 0.043)	0.105	−0.039 (−0.062, −0.016)	0.001*	0.003*

Model 1 unadjusted; Model 2 was adjusted for age, education and apo ε4 status; model 3 was adjusted for the same variables as in model 2 and further adjusted for CMBs and lnWMH; model 4 was adjusted for the same variables as in model 3 and further adjusted for CSF p-tau and Aβ42/40. *p < 0.0125. TBI, traumatic brain injury; CSO, centrum semiovale; PVS, perivascular spaces; RAVLT, rey auditory verbal learning test.

**FIGURE 3:**
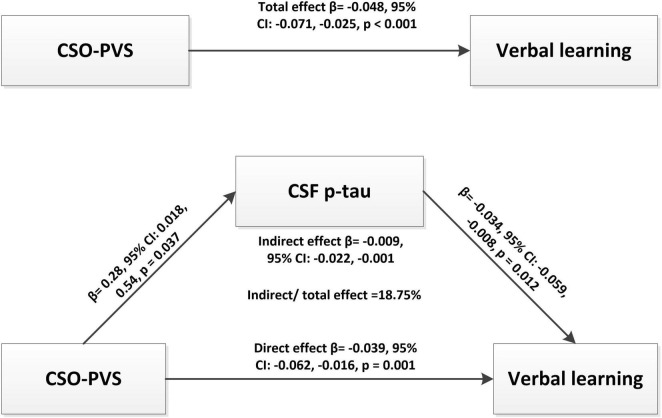
Mediation analysis revealed that CSF p-tau had a significant indirect effect (β = -0.009, 95% CI: -0.022 -0.001, *p* = 0.001) and mediated 18.75% effect for the relationship between CSO-PVS and verbal memory in traumatic brain injury (TBI) group after adjusting for age, education, apo e4, CMBs, and lnWMH.

Further mediation analysis revealed that CSF p-tau had a significant indirect effect (β = −0.009, 95% CI: −0.022 −0.001, *p* = 0.001) and mediated 18.75% effect on the relationship between CSO-PVS and verbal memory in TBI group after adjusting for age, education, apo ε4 status, CMBs, and lnWMH (Figure 3).

## Discussion

In our study, we aimed to investigate the distribution characteristics of MRI-visible PVS and its association with cognitive impairment in military veterans with TBI, and whether CSF biomarkers mediate this effect. Our study revealed that CSO-PVS was more common in Vietnam War veterans with a history of TBI and was associated with poor verbal memory, mediated partially by CSF p-tau. Our study suggested that MRI-visible CSO-PVS could be a promising indicator for cognitive impairment and the CSF p-tau may be the missing link between MRI-visible CSO-PVS and cognitive impairment in military veterans with TBI.

With the wide use of MRI, PVS is frequently detected in clinical practice. MRI-visible PVS has been reported to occur in 100% normal elderly (33–36) and several studies found an increased number of MRI-visible PVS in TBI (19–21). A previous small study revealed that TBI subjects had an asymmetric distribution of MRI-visible PVS, but no higher MRI-visible PVS number compared with healthy controls (37). Compared with military control subjects, Vietnam War veterans with a history of TBI had increased PVS only in CSO region but not in the BG region in our study. Our study suggested that CSO-PVS may be more easily affected by TBI than BG-PVS. The underlying pathophysiological mechanism of MRI-visible PVS in TBI was poorly investigated. One possible explanation was that the cortical surface, and cerebral white matter were commonly affected areas by the TBI-related shear-strain injury, which could stretch perivascular space and finally lead to MRI-visible PVS.

Interestingly, high MRI-visible CSO-PVS number was associated with poor verbal memory in TBI group but not military control group after adjusting for clinical characteristics and other neuroimaging markers. Previous studies have revealed associations of MRI-visible CSO-PVS with Alzheimer’s disease (38) and longitudinal cognitive impairment (39). Our study suggested that there may be an independent mechanism for the association between MRI-visible CSO-PVS and verbal memory in veterans with TBI, possibly by impaired glymphatic system. A recent study revealed associations of PVS in the posterior cingulate, fusiform, and postcentral with somatization symptoms in mTBI (40). Furthermore, TBI and poor sleep had an enhanced interaction effect on PVS indicating a possible role of the glymphatic system (41, 42). However, these mechanisms may be weak in subjects without TBI. A recent study also found no association between MRI-visible CSO-PVS and cognitive tests in cognitively unimpaired (43). Our study demonstrated that MRI-visible CSO-PVS could be a promising imaging biomarker for verbal memory impairment in veterans with TBI.

In our study, higher CSF p-tau was found in military veterans with TBI and CSF p-tau mediated partially the relationship between MRI-visible CSO-PVS and verbal memory. A recent study using the same database also revealed higher CSF p-tau in veterans with TBI than military control (44). The higher CSF p-tau might partially reflect the insufficient function of brain glymphatic system. A previous animal study suggested chronic impairment of brain glymphatic system and tau aggregation after TBI (18). The excessive tau protein might stack in the PVS causing the enlargement of PVS which would be visible on MRI, and the malfunction of PVS would slow the clearance of brain waste, suggesting a feed-forward mechanism. The heavy tau burden would further cause damage to the brain neurons and finally lead to cognitive impairment. A recent study also revealed blood p-tau was associated with cognitive impairment in veterans with a history of TBI (45). Therefore, CSF p-tau may be the missing link between MRI-visible CSO-PVS and cognitive impairment. However, a recent study about mild cognitive impairment found no association between PVS volume fraction and CSF p-tau (46). We speculated that tau pathology may be more prominent in cognitive impairment with TBI. Glymphatic failure has been suggested as a final common pathway to dementia and may constitute a therapeutically targetable pathway (47). Our findings add to the evidence linking MRI-visible PVS to cognitive impairment through possible glymphatic system in veterans with TBI.

Our study had several limitations. First, the number of subjects with a history of TBI was small (*n* = 55). Studies using large sample are needed to replicate our results. Second, due to the cross-sectional nature of this study, the exact order of causal effects between MRI-visible CSO-PVS, CSF p-tau and cognitive impairment cannot be determined. Further longitudinal studies are required to better assess the causal effects. Third, the MRI scanners used in this study were from two different vendors (GE, or Siemens). However, the MRI scanning protocol and parameters were almost identical. To reduce the influence of scanners in the detectability of PVS, we also took MRI scanners as a covariate into the ANCOVA analysis of PVS. Fourth, the detectability of PVS on MRI does not necessarily mean that they are the enlargement of perivascular spaces. Future correlational study with the histopathological changes in autopsy study or animal study is needed to figure out the comprehensive mechanism of MRI-visible CSO-PVS in TBI.

Furthermore, we only used visual rating scales to evaluate PVS, which was observer-dependent. There have been many semi-automatic or automatic methods to quantify PVS (48–55). However, these methods also had limitations and restrictions like 3D MRI sequence in 7T MRI (51, 53, 54). Imperfection of the brain parcellation could affect automated quantification (48). Closely aligned multimodal data sets is needed and there is uncertainty in volume estimates resulting from partial volume effects present in radiologic methods (49). Although called “automatic,” these methods still require some manual intervention (50), and visual checking and editing is likely to be needed in complex cases (52). As these methods were still not applied in clinical practice, we only used visual rating measurement. The inter-rater and intra-rater reliability for the rating of PVS was excellent in our study and could be applied easily in practical clinical practice.

In conclusion, our study found that MRI-visible CSO-PVS was more common in Vietnam War veterans with a history of TBI and was associated with poor verbal memory, mediated partially by CSF p-tau. Our study suggested that MRI-visible CSO-PVS could be a promising indicator for cognitive decline and the CSF p-tau may be the missing link between MRI-visible CSO-PVS and cognitive impairment in military veterans with TBI.

## Data Availability Statement

The datasets presented in this study can be found in online repositories. The names of the repository/repositories and accession number(s) can be found below: http://adni.loni.usc.edu/.

## Ethics Statement

The studies involving human participants were reviewed and approved by the Institutional Review Boards of all participating ADNI sites. The patients/participants provided their written informed consent to participate in this study.

## Author Contributions

M-LW and Y-HL: study concept and design. M-LW, D-XY, and ZS: analysis and interpretation of data and drafting of the manuscript. M-LW, D-XY, Q-QZ, P-YL, XW, and W-BL: statistical analysis and critical revision of the manuscript. Y-HL: administrative, technical and material support, and study supervision. All authors gave final approval of the version published.

## Conflict of Interest

The authors declare that the research was conducted in the absence of any commercial or financial relationships that could be construed as a potential conflict of interest.

## Publisher’s Note

All claims expressed in this article are solely those of the authors and do not necessarily represent those of their affiliated organizations, or those of the publisher, the editors and the reviewers. Any product that may be evaluated in this article, or claim that may be made by its manufacturer, is not guaranteed or endorsed by the publisher.

## Footnotes

1
http://adni.loni.usc.edu

## References

[1] Dewan MC Rattani A Gupta S Baticulon RE Hung Y-C Punchak M Estimating the global incidence of traumatic brain injury. *J Neurosurg.* (2018) 130:1080–97. 10.3171/2017.10.JNS17352 29701556

[2] Langlois JA Rutland-Brown W Wald MM. The epidemiology and impact of traumatic brain injury: a brief overview. *J Head Trauma Rehabil.* (2006) 21:375–8. 16983222 10.1097/00001199-200609000-00001

[3] Das RR Moorthi RN. Traumatic brain injury in the war zone. *N Engl J Med.* (2005) 353:633–4. 10.1056/NEJM200508113530621 16093477

[4] Kaup AR Peltz C Kenney K Kramer JH Diaz-Arrastia R Yaffe K. Neuropsychological profile of lifetime traumatic brain injury in older veterans. *J Int Neuropsychol Soc.* (2017) 23:56–64. 10.1017/S1355617716000849 27697088 PMC5243167

[5] Lippa SM French LM Bell RS Brickell TA Lange RT. United States military service members demonstrate substantial and heterogeneous long-term neuropsychological dysfunction after moderate, severe, and penetrating traumatic brain injury. *J Neurotrauma.* (2020) 37:608–17. 10.1089/neu.2019.6696 31559904

[6] Barnes DE Byers AL Gardner RC Seal KH Boscardin WJ Yaffe K. Association of mild traumatic brain injury with and without loss of consciousness with dementia in US military veterans. *JAMA Neurol.* (2018) 75:1055–61. 10.1001/jamaneurol.2018.0815 29801145 PMC6143113

[7] McKee AC Robinson ME. Military-related traumatic brain injury and neurodegeneration. *Alzheimers Dementia.* (2014) 10(3 Suppl.):S242–53. 10.1016/j.jalz.2014.04.003 PMC4255273 24924675

[8] Mohamed AZ Cumming P Srour H Gunasena T Uchida A Haller CN Amyloid pathology fingerprint differentiates post-traumatic stress disorder and traumatic brain injury. *Neuroimage Clin.* (2018) 19:716–26. 30009128 10.1016/j.nicl.2018.05.016 PMC6041560

[9] Mohamed AZ Cumming P Götz J Nasrallah F. Tauopathy in veterans with long-term posttraumatic stress disorder and traumatic brain injury. *Eur J Nucl Med Mol Imaging.* (2019) 46:1139–51. 30617964 10.1007/s00259-018-4241-7 PMC6451714

[10] Wardlaw JM Benveniste H Nedergaard M Zlokovic BV Mestre H Lee H Perivascular spaces in the brain: anatomy, physiology and pathology. *Nat Rev Neurol.* (2020) 16:137–53. 32094487 10.1038/s41582-020-0312-z

[11] Kwee RM Kwee TC. Virchow-robin spaces at MR imaging. *Radiographics.* (2007) 27:1071–86. 17620468 10.1148/rg.274065722

[12] Doubal FN MacLullich AMJ Ferguson KJ Dennis MS Wardlaw JM. Enlarged perivascular spaces on MRI are a feature of cerebral small vessel disease. *Stroke.* (2010) 41:450–4. 20056930 10.1161/STROKEAHA.109.564914

[13] Iliff JJ Wang M Liao Y Plogg BA Peng W Gundersen GA A paravascular pathway facilitates CSF flow through the brain parenchyma and the clearance of interstitial solutes, including amyloid β. *Sci Transl Med.* (2012) 4:147ra11. 10.1126/scitranslmed.3003748 22896675 PMC3551275

[14] Rasmussen MK Mestre H Nedergaard M. The glymphatic pathway in neurological disorders. *Lancet Neurol.* (2018) 17:1016–24. 30353860 10.1016/S1474-4422(18)30318-1 PMC6261373

[15] Venkat P Chopp M Zacharek A Cui C Zhang L Li Q White matter damage and glymphatic dysfunction in a model of vascular dementia in rats with no prior vascular pathologies. *Neurobiol Aging.* (2017) 50:96–106. 10.1016/j.neurobiolaging.2016.11.002 27940353 PMC5209254

[16] Vilor-Tejedor N Ciampa I Operto G Falcón C Suárez-Calvet M Crous-Bou M Perivascular spaces are associated with tau pathophysiology and synaptic dysfunction in early Alzheimer’s continuum. *Alzheimers Res Ther.* (2021) 13:135. 10.1186/s13195-021-00878-5 34353353 PMC8340485

[17] Park YW Shin N-Y Chung SJ Kim J Lim SM Lee PH Magnetic resonance imaging-visible perivascular spaces in basal ganglia predict cognitive decline in Parkinson’s disease. *Mov Disord.* (2019) 34:1672–9. 10.1002/mds.27798 31322758

[18] Iliff JJ Chen MJ Plog BA Zeppenfeld DM Soltero M Yang L Impairment of glymphatic pathway function promotes tau pathology after traumatic brain injury. *J Neurosci.* (2014) 34:16180–93. 10.1523/JNEUROSCI.3020-14.2014 25471560 PMC4252540

[19] Inglese M Bomsztyk E Gonen O Mannon LJ Grossman RI Rusinek H. Dilated perivascular spaces: hallmarks of mild traumatic brain injury. *AJNR Am J Neuroradiol.* (2005) 26:719–24. 15814911 PMC7977096

[20] Inglese M Grossman RI Diller L Babb JS Gonen O Silver JMA Clinical significance of dilated Virchow-Robin spaces in mild traumatic brain injury. *Brain Inj.* (2006) 20:15–21. 10.1080/02699050500309593 16403696

[21] Orrison WW Hanson EH Alamo T Watson D Sharma M Perkins TG Traumatic brain injury: a review and high-field MRI findings in 100 unarmed combatants using a literature-based checklist approach. *J Neurotrauma.* (2009) 26:689–701. 10.1089/neu.2008.0636 19335205

[22] Mueller SG Weiner MW Thal LJ Petersen RC Jack CR Jagust W Ways toward an early diagnosis in Alzheimer’s disease: the Alzheimer’s disease neuroimaging initiative (ADNI). *Alzheimers Dementia.* (2005) 1:55–66. 10.1016/j.jalz.2005.06.003 17476317 PMC1864941

[23] Malec JF Brown AW Leibson CL Flaada JT Mandrekar JN Diehl NN The mayo classification system for traumatic brain injury severity. *J Neurotrauma.* (2007) 24:1417–24. 17892404 10.1089/neu.2006.0245

[24] Hansson O Lehmann S Otto M Zetterberg H Lewczuk P. Advantages and disadvantages of the use of the CSF Amyloid β (Aβ) 42/40 ratio in the diagnosis of Alzheimer’s disease. *Alzheimers Res Ther.* (2019) 11:34. 10.1186/s13195-019-0485-0 31010420 PMC6477717

[25] Jack CR Bennett DA Blennow K Carrillo MC Dunn B Haeberlein SB NIA-AA research framework: toward a biological definition of Alzheimer’s disease. *Alzheimers Dement.* (2018) 14:535–62. 10.1016/j.jalz.2018.02.018 29653606 PMC5958625

[26] Hicks R Giacino J Harrison-Felix C Manley G Valadka A Wilde EA. Progress in developing common data elements for traumatic brain injury research: version two–the end of the beginning. *J Neurotrauma.* (2013) 30:1852–61. 10.1089/neu.2013.2938 23725058 PMC3814822

[27] Wardlaw JM Smith EE Biessels GJ Cordonnier C Fazekas F Frayne R Neuroimaging standards for research into small vessel disease and its contribution to ageing and neurodegeneration. *Lancet Neurol.* (2013) 12:822–38. 23867200 10.1016/S1474-4422(13)70124-8 PMC3714437

[28] Maclullich AMJ Wardlaw JM Ferguson KJ Starr JM Seckl JR Deary IJ. Enlarged perivascular spaces are associated with cognitive function in healthy elderly men. *J Neurol Neurosurg Psychiatry.* (2004) 75:1519–23. 15489380 10.1136/jnnp.2003.030858 PMC1738797

[29] Fazekas F Chawluk JB Alavi A Hurtig HI Zimmerman RA. MR signal abnormalities at 1.5 T in Alzheimer’s dementia and normal aging. *AJR Am J Roentgenol.* (1987) 149:351–6. 10.2214/ajr.149.2.351 3496763

[30] Cordonnier C Potter GM Jackson CA Doubal F Keir S Sudlow CLM improving interrater agreement about brain microbleeds: development of the brain observer microbleed scale (BOMBS). *Stroke.* (2009) 40:94–9. 10.1161/STROKEAHA.108.526996 19008468

[31] Hayes AF. *Introduction to Mediation, Moderation, and Conditional Process Analysis: A Regression-Based Approach.* New York, NY: Guilford Press (2013).

[32] Gong L Xu R Liu D Lan L Zhang B Zhang C. The specific impact of apolipoprotein E epsilon 2 on cognition and brain function in cognitively normal elders and mild cognitive impairment patients. *Front Aging Neurosci.* (2020) 11:374. 10.3389/fnagi.2019.00374 32226373 PMC7081769

[33] Zhu YC Dufouil C Mazoyer B Soumaré A Ricolfi F Tzourio C Frequency and location of dilated Virchow-Robin spaces in elderly people: a population-based 3D MR imaging study. *AJNR Am J Neuroradiol.* (2011) 32:709–13. 10.3174/ajnr.A2366 21349956 PMC7965873

[34] Yakushiji Y Charidimou A Hara M Noguchi T Nishihara M Eriguchi M Topography and associations of perivascular spaces in healthy adults: the Kashima scan study. *Neurology.* (2014) 83:2116–23. 10.1212/WNL.0000000000001054 25361776

[35] Piantino J Boespflug EL Schwartz DL Luther M Morales AM Lin A Characterization of MR imaging-visible perivascular spaces in the white matter of healthy adolescents at 3T. *AJNR Am J Neuroradiol.* (2020) 41:2139–45. 10.3174/ajnr.A6789 33033050 PMC7658833

[36] Barisano G Sheikh-Bahaei N Law M Toga AW Sepehrband F. Body mass index, time of day and genetics affect perivascular spaces in the white matter. *J Cereb Blood Flow Metab.* (2021) 41:1563–78. 10.1177/0271678X20972856 33183133 PMC8221772

[37] Duncan D Barisano G Cabeen R Sepehrband F Garner R Braimah A Analytic tools for post-traumatic epileptogenesis biomarker search in multimodal dataset of an animal model and human patients. *Front Neuroinform.* (2018) 12:86. 10.3389/fninf.2018.00086 30618695 PMC6307529

[38] Banerjee G Kim HJ Fox Z Jäger HR Wilson D Charidimou A MRI-visible perivascular space location is associated with Alzheimer’s disease independently of amyloid burden. *Brain.* (2017) 140:1107–16. 10.1093/brain/awx003 28335021

[39] Paradise M Crawford JD Lam BCP Wen W Kochan NA Makkar S Association of dilated perivascular spaces with cognitive decline and incident dementia. *Neurology.* (2021) 96:e1501–11. 33504642 10.1212/WNL.0000000000011537 PMC8032377

[40] Sibilia F Custer RM Irimia A Sepehrband F Toga AW Cabeen RP. Life after mild traumatic brain injury: widespread structural brain changes associated with psychological distress revealed with multimodal magnetic resonance imaging. *Biol Psychiatry Glob Open Sci.* (2022). 10.1016/j.bpsgos.2022.03.004 PMC10382710 37519474

[41] Opel RA Christy A Boespflug EL Weymann KB Case B Pollock JM Effects of traumatic brain injury on sleep and enlarged perivascular spaces. *J Cereb Blood Flow Metab.* (2019) 39:2258–67. 10.1177/0271678X18791632 30092696 PMC6827121

[42] Piantino J Schwartz DL Luther M Newgard C Silbert L Raskind M Link between mild traumatic brain injury, poor sleep, and magnetic resonance imaging: visible perivascular spaces in veterans. *J Neurotrauma.* (2021) 38:2391–9. 10.1089/neu.2020.7447 33599176 PMC8390772

[43] Gertje EC van Westen D Panizo C Mattsson-Carlgren N Hansson O. Association of enlarged perivascular spaces and measures of small vessel and Alzheimer disease. *Neurology.* (2021) 96:e193–202. 33046608 10.1212/WNL.0000000000011046

[44] Clark AL Weigand AJ Bangen KJ Thomas KR Eglit GML Bondi MW Higher cerebrospinal fluid tau is associated with history of traumatic brain injury and reduced processing speed in Vietnam-era veterans: a department of defense Alzheimer’s disease neuroimaging initiative (DOD-ADNI) study. *Alzheimers Dement (Amst).* (2021) 13:e12239. 10.1002/dad2.12239 34692979 PMC8515227

[45] Peltz CB Kenney K Gill J Diaz-Arrastia R Gardner RC Yaffe K. Blood biomarkers of traumatic brain injury and cognitive impairment in older veterans. *Neurology.* (2020) 95:e1126–33. 32571850 10.1212/WNL.0000000000010087 PMC7538225

[46] Sepehrband F Barisano G Sheikh-Bahaei N Choupan J Cabeen RP Lynch KM Volumetric distribution of perivascular space in relation to mild cognitive impairment. *Neurobiol Aging.* (2021) 99:28–43. 10.1016/j.neurobiolaging.2020.12.010 33422892 PMC7902350

[47] Nedergaard M Goldman SA. Glymphatic failure as a final common pathway to dementia. *Science.* (2020) 370:50–6. 10.1126/science.abb8739 33004510 PMC8186542

[48] Sepehrband F Barisano G Sheikh-Bahaei N Cabeen RP Choupan J Law M Image processing approaches to enhance perivascular space visibility and quantification using MRI. *Sci Rep.* (2019) 9:12351. 10.1038/s41598-019-48910-x 31451792 PMC6710285

[49] Boespflug EL Schwartz DL Lahna D Pollock J Iliff JJ Kaye JA MR imaging-based multimodal autoidentification of perivascular spaces (mMAPS): automated morphologic segmentation of enlarged perivascular spaces at clinical field strength. *Radiology.* (2018) 286:632–42. 10.1148/radiol.2017170205 28853674 PMC5790307

[50] Wang X Valdés Hernández MDC Doubal F Chappell FM Piper RJ Deary IJ Development and initial evaluation of a semi-automatic approach to assess perivascular spaces on conventional magnetic resonance images. *J Neurosci Methods.* (2016) 257:34–44. 10.1016/j.jneumeth.2015.09.010 26416614 PMC4666413

[51] Hou Y Park SH Wang Q Zhang J Zong X Lin W Enhancement of perivascular spaces in 7 T MR image using Haar transform of non-local cubes and block-matching filtering. *Sci Rep.* (2017) 7:8569. 10.1038/s41598-017-09336-5 28819140 PMC5561084

[52] Ballerini L Lovreglio R Valdés Hernández MDC Ramirez J MacIntosh BJ Black SE Perivascular spaces segmentation in brain MRI using optimal 3D filtering. *Sci Rep.* (2018) 8:2132. 10.1038/s41598-018-19781-5 29391404 PMC5794857

[53] Zong X Park SH Shen D Lin W. Visualization of perivascular spaces in the human brain at 7T: sequence optimization and morphology characterization. *Neuroimage.* (2016) 125:895–902. 10.1016/j.neuroimage.2015.10.078 26520772

[54] Park SH Zong X Gao Y Lin W Shen D. Segmentation of perivascular spaces in 7T MR image using auto-context model with orientation-normalized features. *Neuroimage.* (2016) 134:223–35. 10.1016/j.neuroimage.2016.03.076 27046107 PMC4912922

[55] Hernández Mdel C Piper RJ Wang X Deary IJ Wardlaw JM. Towards the automatic computational assessment of enlarged perivascular spaces on brain magnetic resonance images: a systematic review. *J Magn Reson Imaging.* (2013) 38:774–85. 10.1002/jmri.24047 23441036

