# Supplementary material for: MRI-Visible Perivascular Spaces Associated With Cognitive Impairment in Military Veterans With Traumatic Brain Injury Mediated by CSF P-Tau
Source: Front Psychiatry. 2022 Jul 6;13:921203. doi: 10.3389/fpsyt.2022.921203 (PMC9299379; doi:10.3389/fpsyt.2022.921203)
Supplement: Supplementary file 1 [file Data_Sheet_1.PDF]

Supplementary Material

Supplementary table 1 MRI acquisition parameters

Sequences	Manufacturer	Flip Angle	Matrix	Slice thickness	TR (repetition time)	TE (echo time)	TI (inversion time)
T1	Siemens	9°	240*256	1.2 mm	2300 ms	3 ms	900 ms
	GE	11°	256*256	1.2 mm	7700 ms	3.1 ms	400 ms
T2	Siemens	150°	228*256	4 mm	4000 ms	78 ms	0 ms
	GE	90°	256*256	4 mm	4000 ms	81 ms	0 ms
FLair	Siemens	150°	256*256	5 mm	9000 ms	90 ms	2500 ms
	GE	90°	256*256	5 mm	11000 ms	151.4 ms	2250 ms
T2-Star	Siemens	20°	256*256	4 mm	650 ms	20 ms	0 ms
	GE	20°	256*256	4 mm	650 ms	20 ms	0 ms

Supplementary table 2 Injury characteristics of the TBI subjects

**TBI characteristics**	**N=55**
**Age in last TBI, (years)**	34.3 (19.6)
**TBI frequency (n, %)**	
One TBI	29 (52.7)
Repeated TBI	26 (47.3)
**TBI severity (n, %)**	
Mild TBI	33 (60.0)
Moderate- Severe TBI	22 (40.0)
**TBI hospitalization (n, %)**	32 (58.2)
**TBI with LOC (n, %)**	41 (74.5)
**TBI with PTA (n, %)**	23 (41.8)
**TBI with AOC (n, %)**	42 (76.4)

TBI=traumatic brain injury; LOC=loss of consciousness; PTA= posttraumatic amnesia; AOC=alteration of consciousness.
